# Transcriptomic and physiological analysis reveals the possible mechanism of ultrasound inhibiting strawberry (*Fragaria* × *ananassa Duch*.) postharvest softening

**DOI:** 10.3389/fnut.2022.1066043

**Published:** 2022-12-01

**Authors:** Junyi Zhang, Hui Jiang, Yutong Li, Shaojia Wang, Bei Wang, Junsong Xiao, Yanping Cao

**Affiliations:** Beijing Advanced Innovation Center for Food Nutrition and Human Health (BTBU), School of Food and Health, Beijing Higher Institution Engineering Research Center of Food Additives and Ingredients, Beijing Technology and Business University, Beijing, China

**Keywords:** ultrasound, strawberry, transcriptome sequencing, postharvest softening, mechanism

## Abstract

Ultrasound effectively inhibited strawberry softening but the mechanism was not clear. In this study, physical data including firmness, soluble pectin (SP) contents, pectin esterase (PE), polygalacturonase (PG) activity and transcriptome sequencing data were analyzed to explore the mechanism of strawberry response to ultrasonic treatment. After 24 days storage, the firmness reduction rate and soluble contents (SP) increased rate of the strawberry treated with ultrasound (25 kHz, 0.15 W/cm^2^) for 3 min decreased 41.70 and 63.12% compared with the control, respectively. While the PG and PE enzyme activities of ultrasound-treated strawberries were significantly lower than control after storage for 18 days. A total of 1,905 diferentially expressed genes (DEGs) were identified between ultrasound-treated and control, with 714 genes upregulated and 1,254 genes downregulated, including 56 genes in reactive oxygen species (ROS), auxin (AUX), ethylene (ETH) and jasmonic acid (JA) signaling pathways. At 0 h, 15 genes including *LOX, JMT, ARP, SKP, SAUR, IAA, ARF*, and *LAX* were significantly upregulated compared with the control group, which means reactive oxygen specie, auxin, ethylene and jasmonic acid-mediated signaling pathway respond to ultrasound immediately. *ERF109, ERF110*, and *ACS1_2_6* downregulated before 2 days storage indicated ethylene signaling pathway was inhibited, while after 2 days, 9 genes including *ERF027, ERF109*, and *ERF110* were significantly upregulated indicating that the response of the ethylene signaling pathway was lagging. Therefore, in strawberry ultrasound enhanced ROS scavenging and activated JA biosynthesis, which acts as a signal for delaying the activation of ET signaling pathway, thus suppressing the activity of pectin-degrading enzymes PE and PG, and ultimately inhibiting postharvest softening.

## Introduction

Strawberry (*Fragaria* × *ananassa Duch*.) is widely appreciated due to its fragrance, deliciousness and read high nutritional value, which is a rich source of vitamins, minerals, amino acids, and biologically active ingredients such as phenolic compounds, flavonoids, and anthocyanins (1). However, postharvest strawberries are highly perishable during transport and storage, because they have thin skin and juicy pulp, thus quite vulnerable to mechanical injury, as well as to fungal pathogens spoilage (2). As a pulp fruit, the firmness is one of the most essential quality attributes, which is in relation to fruit quality, resistance to postharvest diseases, shelf life, transportation capacity, and especially the acceptability of consumers (3).

Fruit softening occurs naturally during ripening, primarily as a consequence of polysaccharides (pectin, hemicellulose and cellulose) metabolism of the cell wall and middle lamella (4–6). Numerous researches have concluded that cellulose-hemicellulose network crosslinked with pectin, *via* non-covalent interactions, contributes to cell wall strength and tissue integrity (5, 7, 8). Among the three carbohydrate polymers, pectin consists of 1,4-linked α-D-galacturonic acid residues predominantly, mostly present in the homogalacturonan (HG) and rhamnogalacturonan I (RG-I) (9), hemicelluloses are composed of xylans, xyloglucans as well as mannans, and cellulose is a homopolymer of β-(1,4)-linked glucose residues (10, 11). The disassembly of the cell wall structure requires the orderly participation of multiple cell wall modifying enzymes and proteins. In strawberries, softening is characterized by increasing pectin solubilization, i.e., an increase in the quantity of pectin loosely bound to the cell wall (12). Pectin degrading enzymes, including pectinesterase (PE, EC 3.1.1.11), polygalacturonase (PG, EC 3.2.1.15) and pectate lyase (PL, EC 4.2.2.2), display different activities in pectin metabolism (5, 13). PE is a hydrolase that demethoxylates homogalacturonans in pectin, provide substrates for PG to hydrolyze demethylated polygalacturonic acid to galacturonic acid, resulting in the increase of soluble pectin (SP)content (4, 14), and PL can furtherly cleave β(1-4) linkages between galacturonosyl residues by β-elimination (15, 16). In metabolism of other cell wall polysaccharides, β-galactosidase (β-Gal, EC 3.2.1.23) removes galactosyl residues in side chains of cell wall polysaccharides (pectin and xyloglucan), cellulase (Cx, EC 3.2.1.4) can degrade hemicellulose and cellulose in cell wall (17), β-xylosidase (β-Xyl) (EC 3.2.1.37) hydrolizes xylooligosaccharides releasing xylose units (5). Besides, endo-1,4-beta-glucanase (EGase, EC 3.2.1.4), xyloglucan endo-transglucosylase/hydrolase (XTH, EC 2.4.1.207, and EC 3.2.1.151) participate in hemicellulose metabolism helping cell wall disassembly (5, 13). In addition, expansins (EXPs) may play an indirect role in pectin degradation. These EXP proteins without catalytic activity can promote non-enzymatic cell wall loosening by binding cellulose and hemicellulose and disrupting non-covalent interactions (14).

As mentioned above, fruit postharvest softening is a complex event that requires the orchestrated participation of a wide variety of cell wall degrading proteins. In this process, multiple signals such as reactive oxygen species (ROS), phytohormones including ethylene (ET), abscisic acid (ABA), jasmonic acid (JA), auxin, brassinosteriods and cytokinins, etc., and a set of transcription factors (TFs) are the organizers (5, 13, 18–20). Low level of ROS can serve as a signal molecule in response to abiotic stress in the biological process of plants (18, 21), and hydrogen peroxide (H_2_O_2_) is an important form of ROS. In recent years, there is increasing evidence that ABA plays an important role in regulating fruit ripening process and participating in softening by promoting pectin degradation, including in strawberry (22–25). ET is considered another important regulator in fruit ripening and softening, accomplished by the regulation of a series of ethylene signal transductions, as well as the effect on gene expression of cell wall-degradation enzymes (26). The results of Villarreal et al. (27) confirmed that ethylene participates in the regulation of strawberry fruit softening. JA, considered as a new type of phytohormone, is reported to regulate the coloring, softening and hormone-related gene expression, thus promoting fruit softening and ripening in strawberry (28). Moreover, recent studies have indicated the involvements of auxins, brassinosteriods and cytokinins in fruit ripening and senescence (20). In addition, TFs also play essential roles in gene regulation during fruit ripening (13).

Power ultrasound (20–100 kHz), propagating in a medium in the form of mechanical vibration (29, 30), has been evaluated as an innovative nonthermal preservation technology for fruits and vegetables (31). Ultrasound technology is considered to be attractive, because it can offer significant advantages over other preservation technologies, such as high reproducibility, low cost, ease of treatment, environmentally friendly, and the removal of residues (32, 33). Previous studies have shown that ultrasound treatment can maintain quality characteristics including texture, color, flavor and antioxidant compounds, inactivate microorganisms, influence enzyme activity in fruits and vegetables (34–38). Cavitation effect caused by ultrasound can provide positive or negative effects on enzyme activities, mechanically, by altering the molecular structure of enzymes and their corresponding with substrates (31, 39–41). On the other hand, the free radicals produced by hydrolysis can interact with amino acid residues and affect the activity of enzymes (29, 31). There has been some evidence of the effect of ultrasound on gene expression in plant, for instance, changes of mRNA transcription in potato exposed to ultrasound were observed (42–44). Noteworthily, a prior study in Arabidopsis exposed to sonication discovered transcriptomic changes, and changes in reactive oxygen species (ROS) scavenging ability, as well as in phytohormonal metabolism (45).

To the best of our knowledge, however, few reports are available on the mechanism involved in ultrasound treatment inhibiting postharvest softening of strawberries. In this study, comprehensive analysis of physiological and transcriptomics were utilized to evaluate the effect of ultrasound on strawberry softening during 4°C storage. Furtherly, a broader systemic perspective on possible physiological and molecular regulatory effect of ultrasound treatment on strawberries softening can be obtained.

## Materials and methods

### Strawberry fruit materials

Strawberry fruits (*Fragaria* × *ananassa Duch*. Cv. “Benihoppe”), an octoploid cultivar were harvested from Da Zi Ran farm (Beijing, China), and were immediately transported to laboratory. Well-formed fresh fruits with similar maturity, size and color were selected and stored at 5°C for a night before the experiments.

### Ultrasound treatment and storage

The selected strawberry fruits were completely immersed in a sonicator bath (JXD-02, Beijing Jinxing Ultrasonic Equipment Technology Co., Ltd., China) filled with deionized water for ultrasonic treatment (Frequency 28 kHz, Power 0.15 W/cm^2^, Time 3 min), the surface of water in the bath was kept at the same level and water temperature (15 ± 1°C) was maintained using a low-temperature thermostatic water bath (DC-2006, Ningbo Scientz Biotechnology Co., Ningbo, China). The ultrasound parameter was derived from the orthogonal design experiment in our previous research. Control strawberries samples were subjected to the same conditions and completely immersed in deionized water for the same time as the U treatment. After the treatment, all fruits were dried with absorbent paper and then placed in polyethylene terephthalate (PET) trays (15 fruit per tray) immediately. Later, packaged fruits were stored at 5 ± 1°C and 75–85% RH for 24 d.

### Experimental design

Strawberry fruits after treatments were randomly sampled at different storage time (0 h, 12 h, 1 d, 2 d, 3 d, 6 d, 9 d, 12 d, 15 d, 18 d, 21 d, 24 d) for measurements of hardness, soluble pectin content (SP), PE and PG activity. Among these physiological indicators, fruit samples were homogenized and stored at −20°C after quick-frozen in liquid nitrogen for the determination of PE and PG activity, all the other indicators were measured using fresh samples. Two trays of strawberries were used to measure ethylene production. Signal substance H_2_O_2_ content was measured at 0 h, 3 h, 6 h, 9 h, 12 h, 1 d, 2 d, 3 d, 6 d, 9 d, 12 d, 15 d, 18 d, 21 d, 24 d. Due to the decay of strawberries at 24 d, the ethylene production was no longer measured, and the other time points for ethylene measurement was the same as the H_2_O_2_ content.

Strawberry samples in U and CK group were randomly selected at 0 h, 3 h, 6 h, 9 h, 12 h, 1 d, 2 d, 3 d, 6 d, 9 d during storage, and then the samples were pulped and frozen with liquid nitrogen to store at −80°C for transcriptome sequencing.

Three biological replicates were set in physiological and transcriptome sequencing experiments.

### Firmness and soluble pectin content

Firmness was measured by a digital sclerometer (GY-4, Hangzhou Lvbo Instrument Co., Ltd., China) with 7.9 mm diameter probe, and the actual firmness was twice the shown maximum penetration force (N). Two opposite sides of fruit along the main axis were measured, and five strawberries from each treatment were randomly selected.

Extraction and assay of soluble pectin content was performed according to Cao et al. (46). Strawberry fruit (1.0 g) was prepared as alcohol-insoluble material after repeated washing with 95% ethanol for 4 times. Then, the washed material was incubated in 50°C water for 30 min to obtain the soluble pectin extract. The soluble pectin content was determined with carbazole methods colorimetrically using a microplate spectrophotometer (Infinite 200 PRO, Tecan, Switzerland). Galacturonic acid was used as a calibration standard, and soluble pectin content was expressed as galacturonic acid equivalents (GaE).

### Measurement of PE and PG activity

Extraction and measurement of PE (E 3.1.1.11) was determined by the method of Vicente et al. (47) with modifications. Frozen strawberry pulp (5 g) was homogenized with 15 ml of 1 M NaCl solution containing 1% (w/v) polyvinylpolypyrrolidone (PVPP). The mixture was stored at 4°C for 2 h, and then centrifuged at 4°C and 10,000 × g for 10 min. The pH of supernatant was adjusted to 7.5 with 0.1 M NaOH, the resulting enzymatic extract was used for assaying PE activity. Substrate mixture containing 2.8 mL 1% (w/v) partially methylated (≥85% esterified) citrus pectin solution pH = 7.5, 750 μL 30 mM potassium phosphate buffer (PH = 7.5) containing 0.01% (w/v) bromothymol blue. Reaction-mixture after adding 500 μL PE extract was mixed immediately and used for spectrophotometric measuration (UV spectrophotometer, UVmini-1240, Shimadzu Corporation, Japan) every 20 s during 3 min at 620 nm. Calibration curve was built using different concentration of galacturonic acid (pH = 7.5) as standard. PE activity was expressed as μmol of demethylgalacturonic acid (GalA) produced per second and per kilogram of fruit (μmol·s^−1^·kg^−1^).

The methodology to measure PG (E 3.2.1.15) activity was adapted from Karakurt and Huber (48). Frozen strawberry pulp (10 g) was homogenized with 20 mL of 95% ethanol, placed at 4°C for 10 min, and then centrifuged at 8,000 rpm for 10 min. The precipitate was added with 10 mL 80% ethanol, placed at 4°C for 10 min, and then centrifuged at 8,000 rpm for 5 min. Then, the precipitate was extracted with 5 mL of buffer (50 mM sodium acetate/acetic acid pH 5.5) containing 1.8 M NaCl for 20 min and then centrifuged at 8,000 rpm for 5 min. The supernatant was adjusted to 10 mL, which was the PG extract for activity determination. All the steps were done at 4°C. Reaction mixtures for the determination of PG activity, using polygalacturonic acid as substrate (Shanghai Yuanye Biotechnology Co., Ltd., China.), consisted of 250 μL of the enzymic extract and 250 μL of 10 g/L polygalacturonic acid in 500 μL of 50 mM, pH 5.5 acetic acid-sodium acetate buffer, were incubated for 1 h at 37°C. Enzymic product galacturonic acid was determined by the 3, 5-dinitrosalicylic acid (DNS) method (49), and absorbance at 540 nm was measured on microplate spectrophotometer (Infinite 200 PRO, Tecan, Switzerland). Calibration curve was built using different concentration of galacturonic acid (pH = 7.5) as standard. PG activity was expressed as μg of D-(+)-galacturonic acid produced per hour and per gram of fruit (μg·h^−1^·g^−1^).

### Determination of H_2_O_2_ and ethylene production

H_2_O_2_ was assayed using the method of Brennan and Frenkel (50) with slight modifications. In brief, pulped strawberry (4 g) mixed with 6 mL acetone, and then centrifuged at 8,000 rpm 4°C for 5 min. The supernatant was made up to 10 mL with acetone, which was the H_2_O_2_ extract. One milliliter H_2_O_2_ extract was reacted with 0.1 mL 5% titanium sulfate-sulfuric acid (2 M) and 0.2 mL concentrated ammonia. The precipitate after centrifugation was washed by acetone and finally dissolved in 1 mL 2 M H_2_SO_4_. The absorbance at 415 nm was recorded and the H_2_O_2_ content results was expressed as μmol g^−1^ FW.

Ethylene production was measured as described by Mditshwa et al. (51) with slight modifications. Briefly, five fruits per replicate were used to measure ethylene production. Fruits was weighed using an electronic balance with an accuracy of 0.01 g, and thereafter enclosed in a 400 mL airtight glass jar for 1 h at 20°C. For each ethylene measurement, three jars also were used. SCS56 ethylene analyzer (Storage Control Systems, Inc., USA) was used for ethylene measurements and the results were expressed as μL·kg^−1^·h^−1^.

### RNA extraction and transcriptome sequencing

Total RNA from strawberry samples was extracted using OmniPlant RNA Kit (DNase I) (ComWin Biotech Co., Ltd., China) according to the manufacturer's instruction. Transcriptome sequencing was performed by Shanghai Majorbio Biopharm Technology Co., Ltd. (Shanghai, China). The cDNA library was constructed using the TruSeq™ RNA sample preparation kit (Illumina Inc.). Briefly, 1 μg of total qualified RNA was used for poly-A based mRNA enrichment selection using Oligo (dT) magnetic beads, and then fragmentation buffer was added to randomly interrupt the enriched complete mRNA, small fragments of about 300 bp were separated by bead screening. Under the action of reverse transcriptase and random hexamers (six-base random primers), reverse transcription to synthesize one-strand cDNA was performed using mRNA as template, and then followed by double stranded cDNA synthesis. Double stranded cDNA was end repaired adding “A” tails to the 3′ end for adaptor ligation. Finally, transcriptome sequencing was performed on the Illumina Novaseq 6000 platform with 150 bp paired-end sequencing length. Subsequent bioinformatics analysis was based on clean, high-quality reads obtained by quality control. All raw sequence read data were submitted in the NCBI Sequence Read Archive (SRA, http://www.ncbi.nlm.nih.gov/Traces/sra) database with the accession number PRJNA613956.

### Bioinformatics analysis

The clean data were mapped to the octoploid strawberry reference genome (https://www.rosaceae.org/species/Fragaria_x_ananassa/genome_v1.0.a1) using HISAT2 (52, 53). All genes were annotated in GO, KEGG, COG, NR, Swiss-Prot, and Pfam databases. Gene expression quantitative analysis was carried out using RSEM with TPM (Transcripts Per Million reads) as the quantitative indicator (54). DESeq2 software was used to analyze the differences between different samples and treatment groups (55). Differentially expressed genes (DEGs) were identified when |log_2_ fold change| ≥ 1 and *p*-adjust < 0.05 using the Benjamin-Hochberg multiple hypothesis test correction method (56). KEGG enrichment analysis of the DEGs were performed using Goatools software base on R script, KEGG pathway was considered to be significantly enriched in the gene set when *p*-adjust < 0.05 in Fisher test.

### qRT-PCR validation

In order to confirm the results of the transcriptome sequencing, 9 genes related to ROS, ethylene, auxin signaling pathways, MAPK signal transduction pathway, and fruit softening metabolic pathways, were selected for qRT-PCR (quantitative real-time PCR) analysis. Primer sequence were designed by Primer Premier 5 and their specificity were validated. Synthetic primers were purchased from Synbio Technology Co., Ltd. (China) and shown in Supplementary Table S1. The reference gene *FaActin* primers were F: 5′-GGTGACGAGGCTCAATCCAA-3′, R: 5′-GGGCAACACGAAGCTCATTG-3′.

cDNA was synthesized from quantified total RNA by HiScript^®^ QRT SuperMix for qPCR (+gDNA wiper) (Vazyme Biotech Co., Ltd., Nanjing, China) according to the manufacturer's instruction. Synthesized cDNA (50 ng/μL) which were frozen at −20°C, were diluted 10-fold used as the template for qRT-PCR. qRT-PCR was performed using SuperReal PreMix Plus (SYBR Green) (Tiangen Biotech Co., Ltd., China) in CFX96 Touch™ Real-Time PCR Detection System (Bio-Rad Laboratories, USA). The 10 μL reaction mixture contained 0.3 μL of forward primer (5 μM), 0.3 μL reverse primer (5 μM), 5 ng cDNA template, 5 μL 2 × SuperReal Premix Plus (SYBR Green), and 3.4 μL water. The qRT-PCR conditions were as follows: 15 min at 95°C, followed by 40 cycles of 10 s at 95°C and 20 s at 55°C, and 20 s at 72°C. The melting curves were measured from 65 to 95°C at 0.5°C increments. Relative expression levels of target gene were normalized to internal control *FaActin*, with CK_0h sample as the benchmark, and were calculated by 2^−ΔΔCt^ method (57). For each gene analyzed, a template-free negative control was included in each run, and three independent biological replicates were made for each gene in each sample.

### Statistical analysis

Bioinformatics analysis and graphical presentations of RNA-seq data was completed with online data processing and drawing tools in website (http://www.majorbio.com/). Physiological data was statistically analyzed by independent *t*-tests using SPSS 25 (IBM, Armonk, NY, USA), and differences at *P* < 0.05 level were considered statistically significant. Figures were made by Origin 2019 (Microcal Software Inc., Northampton, MA, USA). The data in the figures were presented as means ± standard errors (SE).

## Results

### Firmness and soluble pectin content after ultrasound treatment

**Figure 1:**
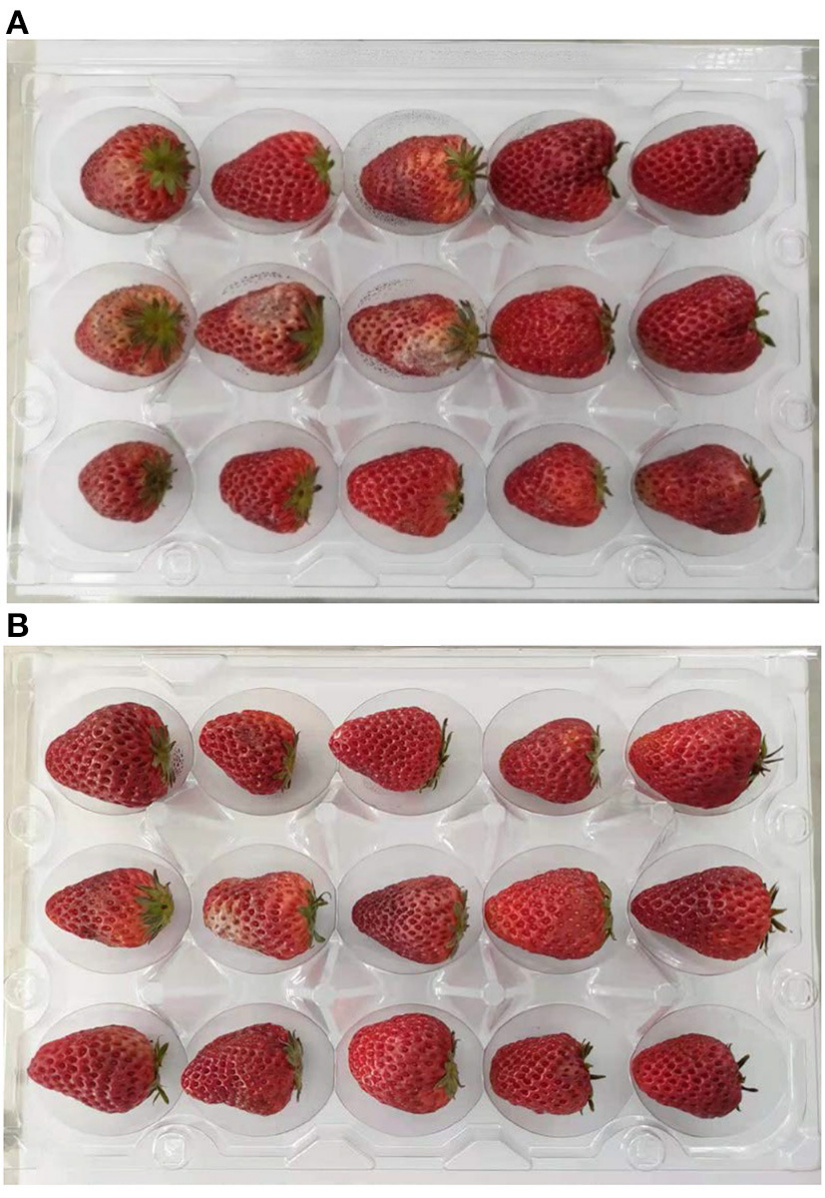
Appearance of the control **(A)** and ultrasound **(B)** groups at 24 d, ultrasound treatment condition was frequency 28 kHz, power 0.15 W/cm^2^, time 3 min.

Fruit firmness is considered to be one of the most important indicators of fruit quality, and it decreases gradually along with fruit ripening and softening during postharvest storage. There was a general decrease in fruit firmness of both the control and ultrasound-treated strawberries during cold storage at 24 d (Figure 1). By the end of storage at day 24, the firmness of the control fruits decreased from 17.36 to 10.52 N, a decrease of 39.40%, while the firmness of the ultrasound-treated fruits decreased only by 22.97%, from 16.98 to 13.08 N. Relative to the control, the firmness of the ultrasound treated strawberries was maintained at a significant (*p* < 0.05) higher level after 18 d. The results showed that the decline in strawberry fruit firmness was inhibited by ultrasound treatment during storage, which was in accordance with the study of Cao et al. (46), Alexandre et al. (58), and Gani et al. (59), finding that ultrasound can effectively maintain the textural quality of strawberry.

**Figure 2:**
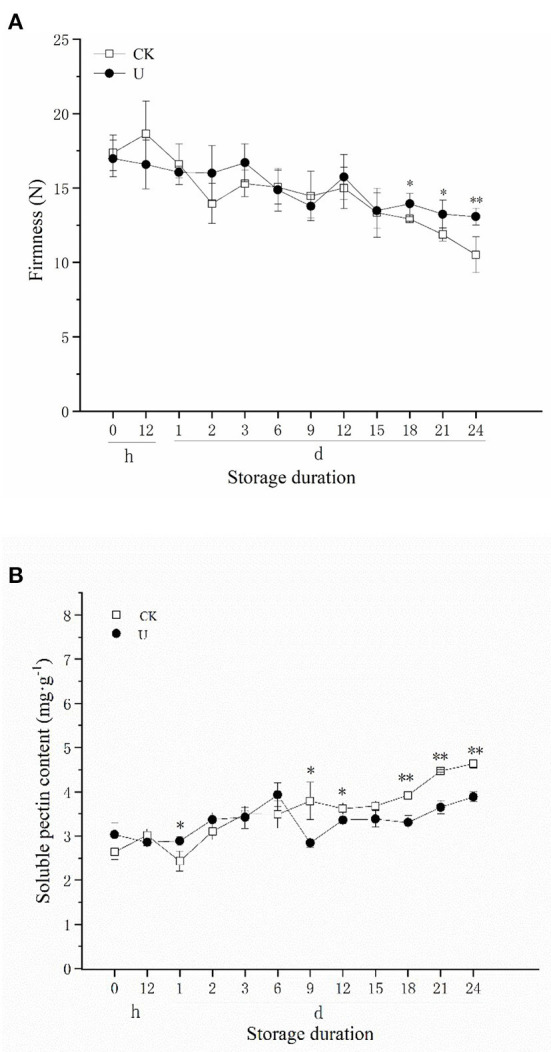
Effect of ultrasound on firmness **(A)** and soluble pectin (SP) content **(B)** in strawberries during storage. Data represent the means ± SE, *n* = 3. According to independent *t*-tests, asterisks indicate significant difference (*P < 0.05, **P < 0.01) between CK and U treatments at the same time point.

The increase of soluble pectin (SP) content in plant cell walls is one of the main reasons for fruit firmness decrease (12). According to Figure 2B, SP content increased with postharvest storage time in two groups. SP content of the control fruits increased from 2.63 to 4.63 mg·g^−1^, an increase of 76.05%, whereas the SP content of the ultrasound- treated fruits increased by 28.05 %, from 3.03 to 3.88 mg·g^−1^. Compared with the control, SP content in ultrasound-treated strawberries was maintained at a significant (*p* < 0.05) lower level generally after 9 d. The results indicated that ultrasound inhibited the production of SP in late storage period, thereby maintained fruit firmness and retarded strawberry fruit softening effectively.

### Activities of PE and PG after ultrasound treatment

**Figure 3:**
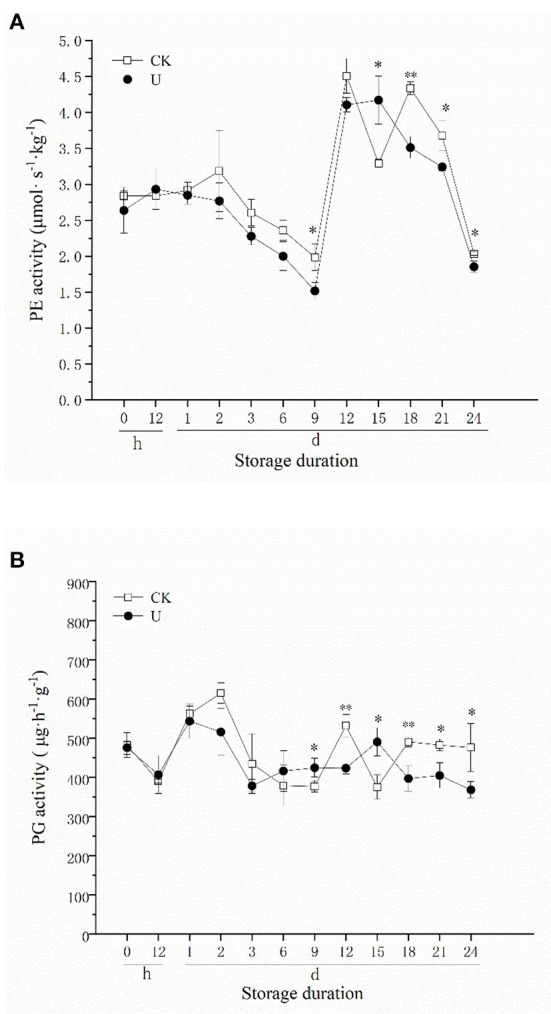
Effect of ultrasound on PE **(A)**, PG **(B)** activities in strawberries during storage. Data represent the means ± SE, *n* = 3. According to independent t-tests, asterisks indicate significant difference (*P < 0.05, **P < 0.01) between CK and U treatments at the same time point.

PE can catalyze the deesterification of polygalacturonic acid in plant cell walls into pectic acid, forming a substrate that facilitates the action of PG, PL and other pectinase, promote the degradation of pectin (60). As shown in Figure 3A, PE activity in ultrasound-treated strawberries showed the same trend as the control. PE activity decreased during the first 9 d, increased rapidly from days 9-12, and then decreased again after day 12. There was no significant difference in PE activity between U and CK group during the first 6 d, and significant differences appeared since 9 d. The PE activity of strawberries in U and CK group reached the highest level at 15 and 12 d respectively, which were 4.17 (U) and 4.51 (CK) μmol·s^−1^·kg^−1^, decreased by 7.54% compared to the control. Moreover, the PE activity of strawberries in U group was significantly lower than CK (*P* < 0.05) after day 18.

PG is one of the main hydrolytic enzymes of plant cell walls, which can further hydrolyze the pectic acid in the fruit cell wall to generate oligomeric galacturonic acid or galacturonic acid, leading to fruit softening. In the two groups, PG activity increased on 0–2 d and then decreased during the first 6 d of storage (Figure 3B) approximately, reached a peak after day 9 and decreased gradually thereafter. There was no significant difference in PG activity between U and CK group during the first 6 d. The PG activity of strawberries in U and CK group peaked on days 15 and 12 respectively, which were 490.20 (U) and 531.41 (CK) μg·h^−1^·g^−1^. Compared with the control, the highest PG activity in ultrasound treated strawberries decreased by 7.75%, and PG activity was also maintained at a significantly lower level since 18 d. These results were similar to the performance of PE activity. In the present study, the time when the PE and PG activity reached the highest level was delayed by ultrasound treatment, and their activities in late storage period were also suppressed by ultrasound.

### H_2_O_2_ production and ethylene production after ultrasound treatment

**Figure 4:**
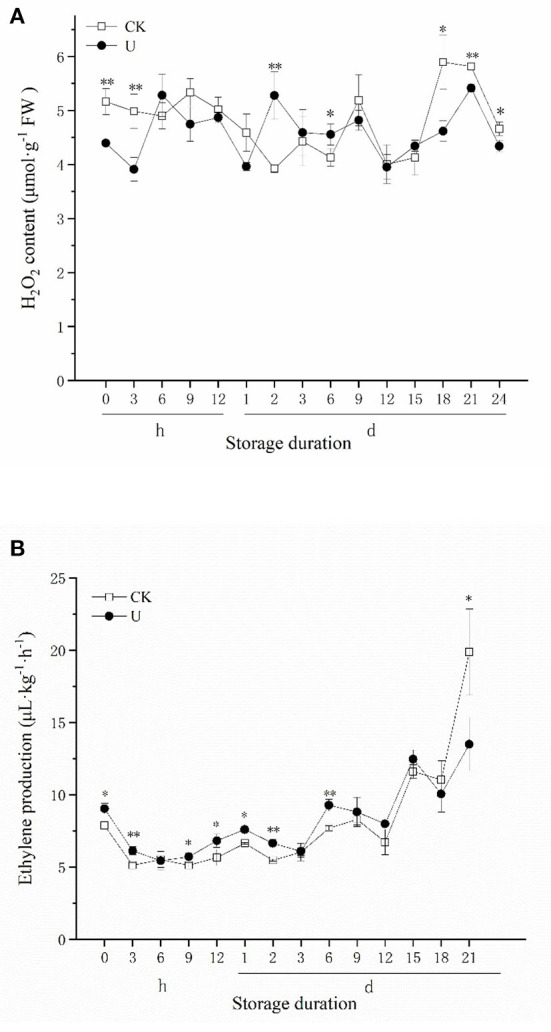
Effect of ultrasound on H_2_O_2_ content **(A)**, ethylene production **(B)** in strawberries during storage. Data represent the means ± SE, *n* = 3. According to independent *t*-tests, asterisks indicate significant difference (*P < 0.05, **P < 0.01) between CK and U treatments at the same time point.

H_2_O_2_ is an important reactive oxygen species (ROS) component, which acts as an important signal substance to regulate the quality of fruits and vegetables during postharvest storage. H_2_O_2_ content was monitored through time for both the control and ultrasound treated strawberries (Figure 4A). It can be seen that the H_2_O_2_ content of ultrasound treated strawberries was significantly lower than that in control at 0 h, 3 h, indicating that ultrasound significantly inhibited H_2_O_2_ production in the early storage period. From 6 h to 15 d, the H_2_O_2_ content almost had no obvious change in two groups, and had no significant difference in strawberries of two groups except on day 2 and 6. Since day 18, the H_2_O_2_ content increased rapidly along with storage time, and the H_2_O_2_ content of the ultrasound group was significantly lower than that of control, indicating that ultrasound reduced the H_2_O_2_ content of the strawberry in the late storage period. The results exhibit that ultrasound affects the ROS metabolism of strawberry fruits mainly by reducing the H_2_O_2_ production in the early and late stages of storage.

Ethylene (ET) is a gaseous hormone that regulates fruit maturation and senescence. Studies have found that ethylene can be used as a signal substance to regulate the texture, color and aroma of non-climacteric fruits. As shown in Figure 4B, during cold storage period after 0 h, the ethylene production of strawberry fruits showed an upward trend. During the first 6 d, the ethylene production of strawberries in the U group was generally significantly higher than that in CK. No significant difference was observed between U and CK group during 9–18 d, while the ethylene production of U group was significantly (*p* < 0.05) lower than CK at 21 d finally.

### Transcriptome sequencing and DEGs analysis

**Figure 5:**
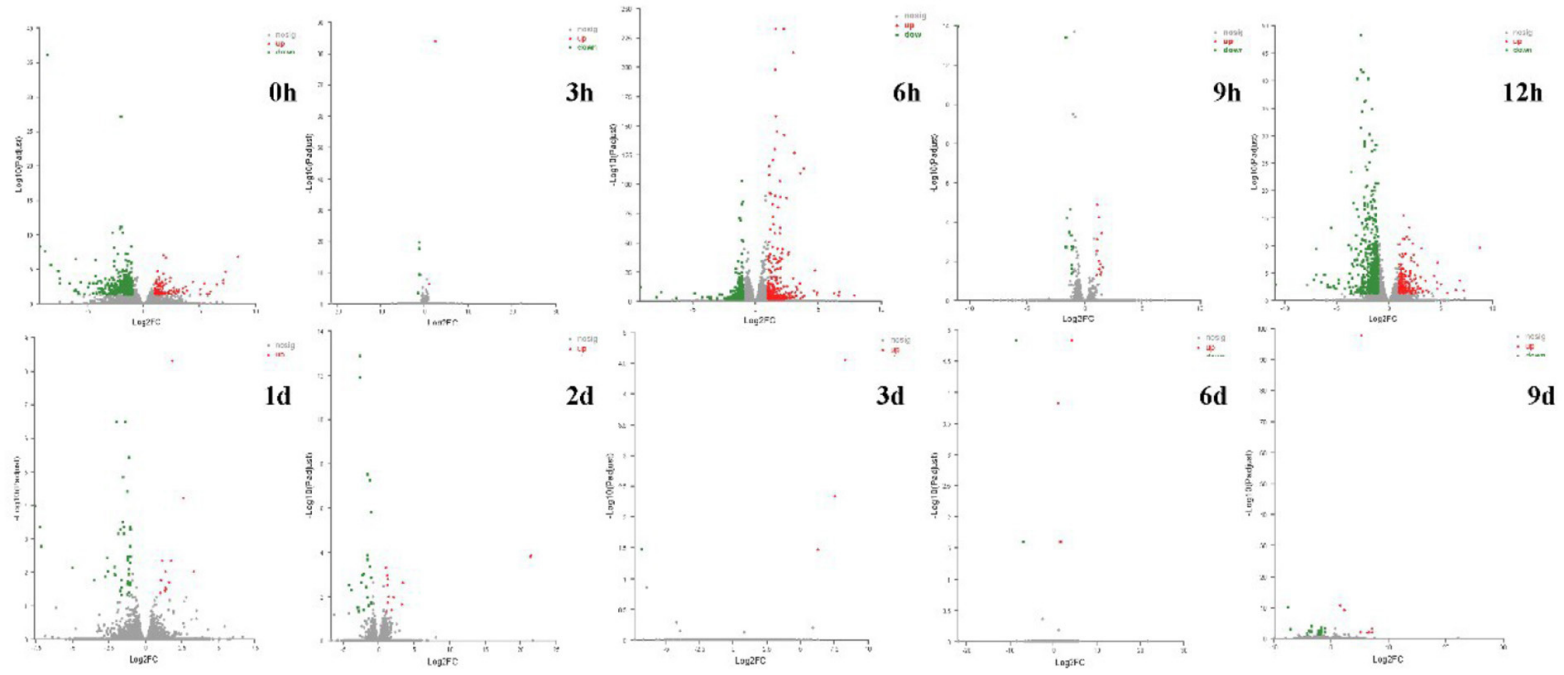
Volcano plot of DEGs (|log2FC|>1, *p*-adjust < 0.05) of strawberry at different time points after treatments. Red and green dots represent the upregulated and downregulated gene transcripts, respectively, and gray dots represent the gene transcripts that expressed differentially but have no significant difference.

A total of 250.05 Gb clean data was obtained from 60 samples, for each sample, above 6.27 Gb clean data (with Q30 > 93.68%) was obtained, and about 85.59–92.18% clean data was mapped to a reference *Fragaria* × *ananassa* genome (v1.0.a1). In total, 85, 634 expressed genes were detected, including 82, 205 known genes and 3, 429 new genes. Differentially expressed genes (DEGs) were identified by comparing the transcripts of the U group samples with CK at the same storage time. A total of 1,905 DEGs were identified at all the 10 time intervals, of these, 714 genes were significantly up-regulated and 1,254 genes were significantly down-regulated. The number of DEGs in samples varies with storage time greatly (Table 1; Figure 5). It was found that 498, 739, and 769 genes differentially expressed at 0, 6, and 12 h after ultrasound treatment, respectively. While the number of DEGs decreased gradually after 12 h, which means that the majority of DEGs (up to 769/1,905) respond to ultrasound treatment in the early stage of storage.

**Table 1:** Numbers of DEGs at different storage time after ultrasound treatment.

**Storage time**	**DEGs**	**Up-regulated DEGs**	**Down-regulated DEGs**
0 h	498	118	380
3 h	6	2	4
6 h	739	375	364
9 h	26	12	14
12 h	769	210	559
1 d	54	12	42
2 d	35	13	22
3 d	4	3	1
6 d	7	4	3
9 d	26	8	18
Total DEGs	1,905	714	1,254

### DEGs GO annotation and KEGG enrichment analysis

**Figure 6:**
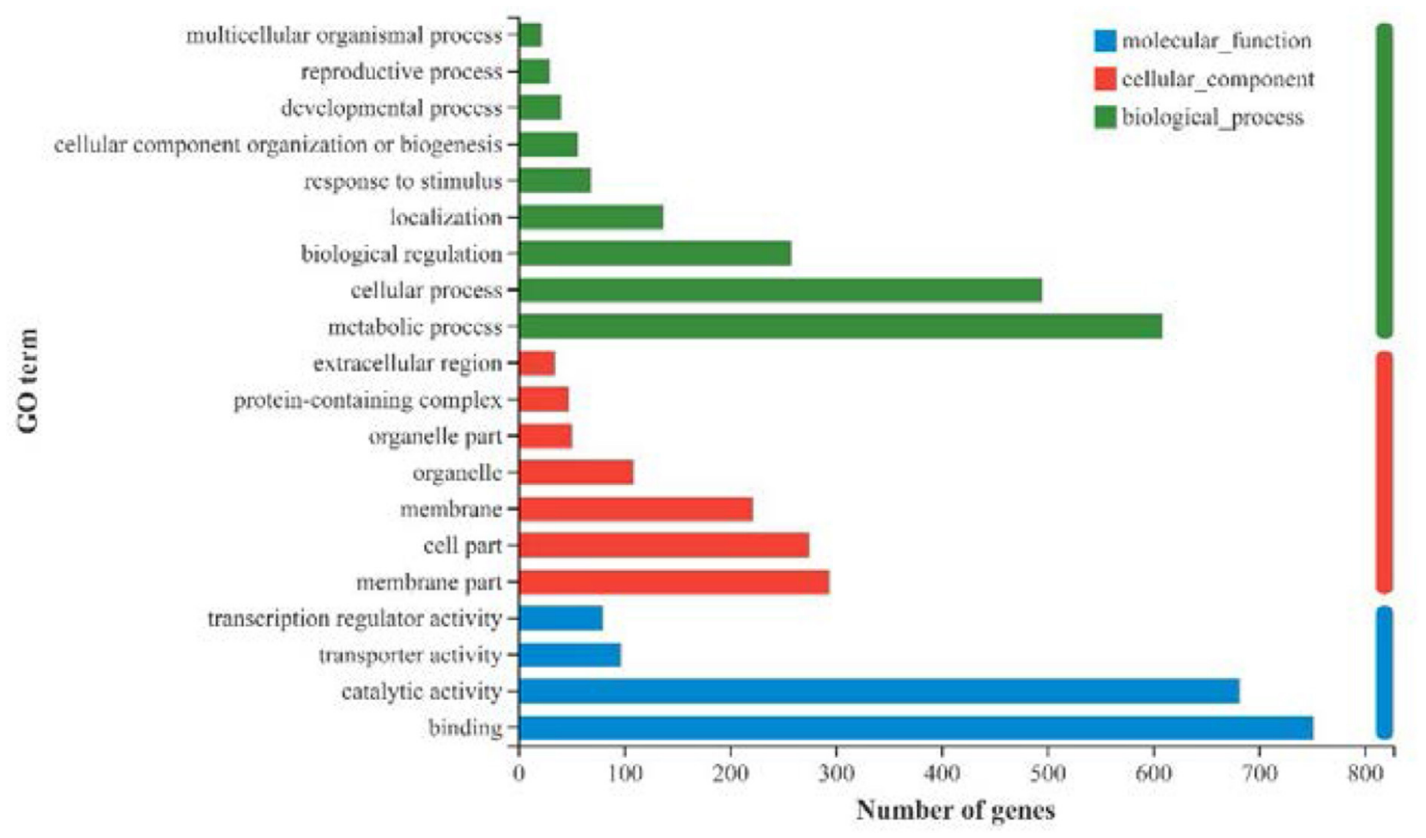
GO annotation of all DEGs (|log2FC|>1, *p*-adjust < 0.05) in strawberry fruit after ultrasound treatment.

All the 1,905 DEGs were subjected to GO functional annotation analysis to determine the biological process, cellular component and molecular function of genes (Figure 6). Among these, 609 genes and 495 genes were associated with metabolic processes and cellular processes in biological process, respectively; 294 genes and 275 genes were associated with membrane part and cell part in cellular component, respectively; 752 genes and 682 genes were associated with binding function and catalytic activity in molecular function, respectively. In addition, there were 258 genes involved in the biological regulation process, and 80 genes owned transcription regulator activity.

**Figure 7:**
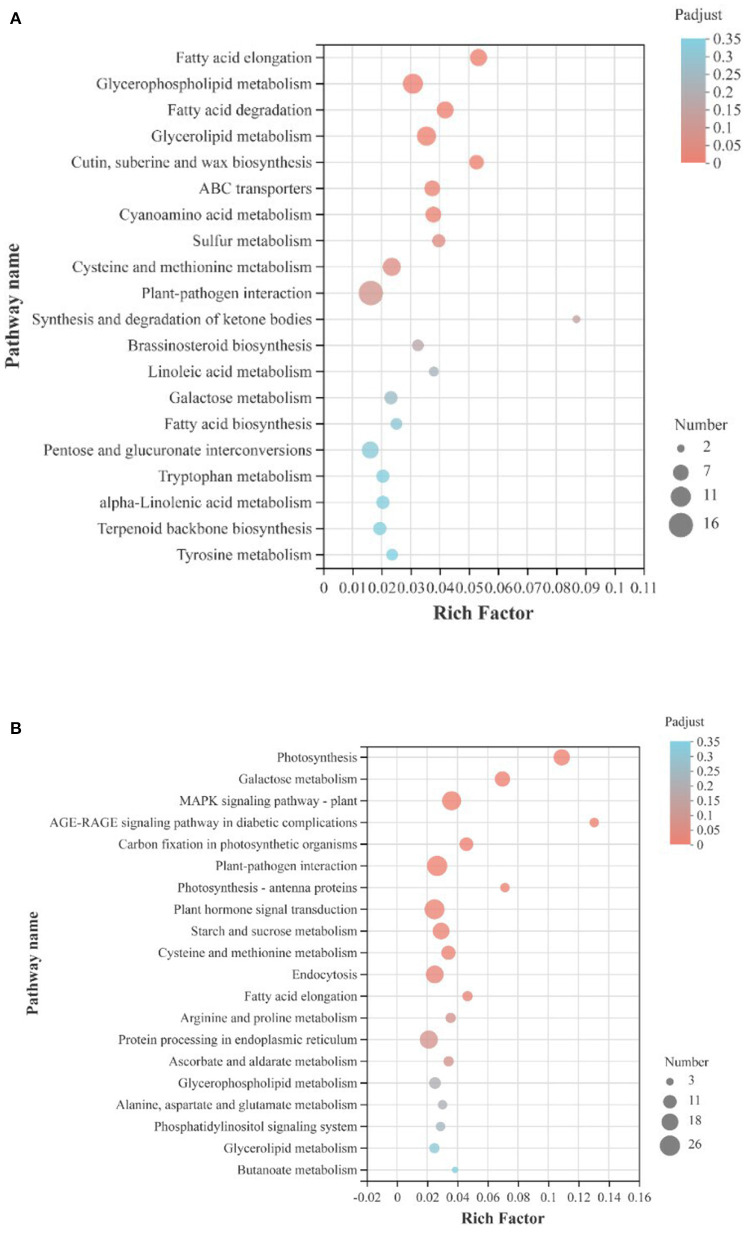
KEGG pathway enrichment analysis of DEGs (|log2FC|>1, *p*-adjust < 0.05) in strawberry fruit after ultrasound treatment. **(A)** Upregulated genes; **(B)** downregulated genes. Rich Factor refers to the ratio of genes enriched in the KEGG pathway to annotated genes. The greater the Rich Factor value, the higher the degree of enrichment. The size of the dot indicates the number of genes in the KEGG pathway, and the color of the dot indicates different p-adjust values. The smaller the *p*-adjust value, the higher the degree of enrichment.

For both up-regulated and down-regulated genes of all 1,905 DEGs, KEGG pathway enrichment analysis was performed (Figure 7). Of the upregulated genes, largest quantity of genes was involved in the plant-pathogen interaction and glycerophospholipid metabolism pathway; of the downregulated genes, largest quantity of genes was also involved in the plant-pathogen interaction metabolism pathway, and followed pathways were plant hormone signal transduction and MAPK signaling pathway. The KEGG pathway enrichment analysis showed that ultrasound treatment had a significant effect on the interaction between strawberry fruit and pathogens. The MAPK signaling pathway can amplify signals through gradual phosphorylation and participate in plant defense responses under stress. The phytohormone signal transduction pathway, MAPK signaling pathway, starch and sucrose metabolic pathway, and galactose metabolic pathway were significantly down-regulated during the first 9 d storage, indicating that the stress response and signal transduction process was milder and metabolism level was lower in ultrasound treated strawberries than in control, which helping to maintain fruit quality.

### Expression profile of genes in ROS, hormone metabolism and signaling pathways and transcription factor family

**Figure 8:**
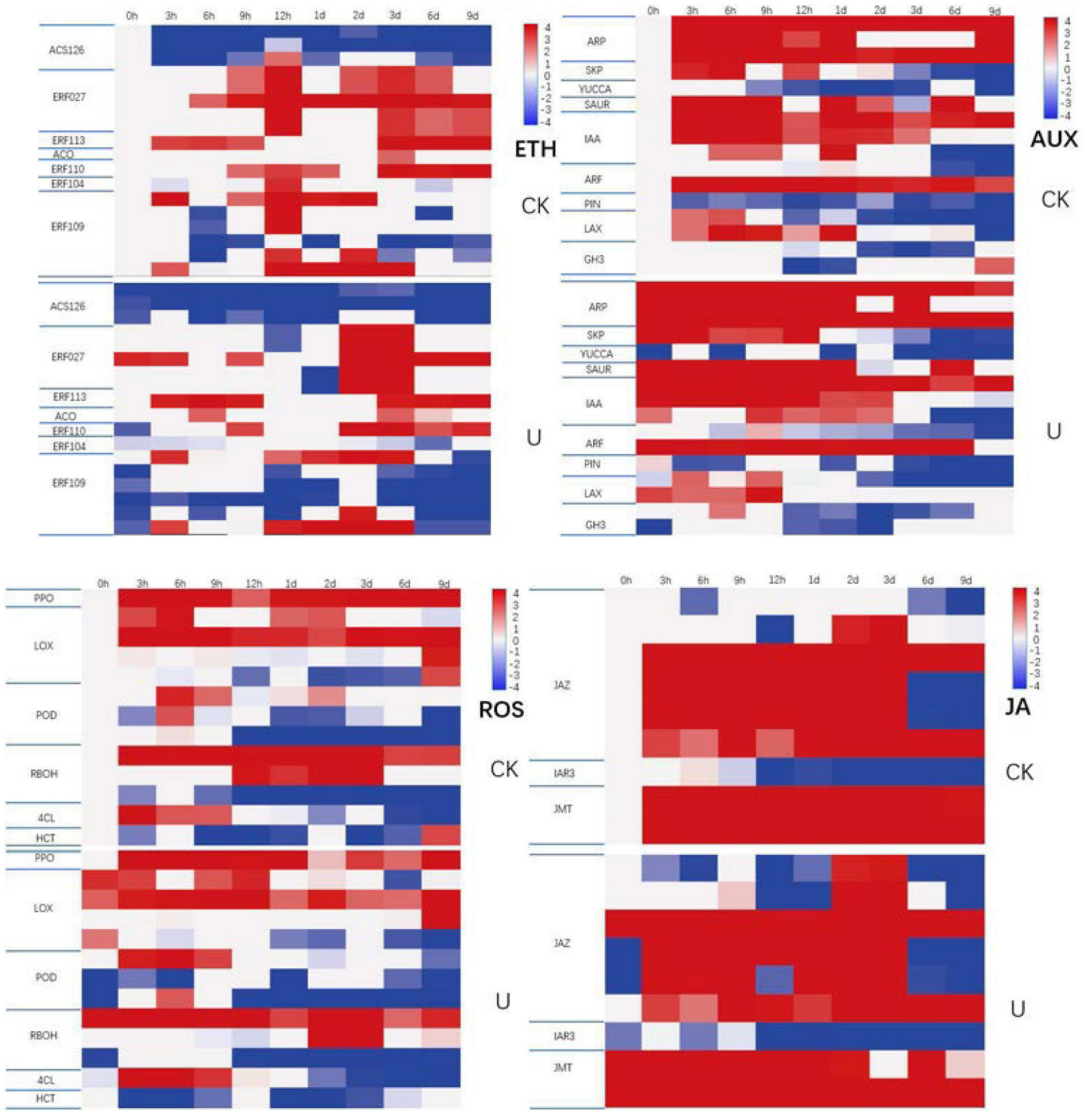
Expression profile of genes involved in ROS, hormone metabolism and signaling pathways and transcription factor during storage as indicated by heatmap. The normalized expression value of each gene in each sample was shown in colored blocks. Red color represents the higher expression level of the gene in the sample, and blue represents the lower expression level. The specific expression level was shown in the upper left color bar. DEGs (differentially expressed genes) were defined with fold change ≥2 and a *p*-adjust < 0.05 as forward descriptions.

A total of 13 genes related to ROS metabolism was investigated (Figure 8). Compared with the control group, four genes differently expressed significantly at 0 h, of which three genes, and encoding LOX were upregulated, and one gene encoding RBOH (NADPH oxidase) (61) was downregulated in the ultrasound-treated group. ROS scavenging of genes encoding LOX was upregulated at 0 h, which was the earliest gene to promote ROS scavenging. Down-regulation of genes encoding RBOH which can catalyze the production of superoxide anion ${\text{(O}}_{2}^{-})$ and H_2_O_2_ showing that ROS production was inhibited at 0 h. At 6 h after ultrasound treatment, six DEGs were observed. Four of which were upregulated, including one gene encoding PPO, one gene encoding 4CL, and two genes encoding POD. Another two genes were downregulated, one gene encoding POD and the other gene encoding HCT. Genes encoding PPO and POD which related to ROS scavenging were partially upregulated, and gene encoding 4CL which can promote phenol synthesis to reduce ROS was upregulated, which mean that ROS production continued to be inhibited and ROS scavenging was enhanced at 6 h. At 12 h, two genes were downregulated, one gene encoding POD and the other gene encoding RBOH. These results indicated that the production of ROS was suppressed and the elimination of ROS was enhanced continuously during the first 12 h after ultrasound treatment.

Nine genes involved in JA metabolism and signaling were differentially expressed during storage (Figure 8). At 0 h, three genes coding JAZ protein which can inhibit JA signaling showed downregulation, resulted in the increasing release of JA signal. At 6 h, the expression of three genes were remarkably regulated. The transcripts of two *JMT* genes which coding JA degrading enzyme jasmonate O-methyltransferase (JMT), were dramatically increased, whereas transcript abundance of *IAR3* which coding IAA-amino acid hydrolase displayed decline, indicating the inhibition of JA synthesis and enhancement of JA degradation to certain degree at 6 h. At 12 h, two *JAZ* genes exhibited significant downregulation, suggested that the release of JA signal was enhanced again at which time.

There were 18 ethylene related genes displayed markedly differences in expression during storage (Figure 8). At 0 h, six ethylene response factor genes were significantly down-regulated which including three genes of *ERF109* and three genes of *ACS1_2_6*. The ethylene signal pathway in the ultrasound treatment group began to respond at 0 h, the control group began to up-regulate from 12 h, and the ultrasound group began to significantly up-regulate at 2 days. The ethylene signal pathway response after ultrasound was prolonged and lagged. Ethylene is synthesized from S-aenosyl methionine (SAM) *via* the intermediate 1-aminocyclopropane-1-carboxylic acid (ACC), in the biosynthesis process, SAM is converted to ACC by ACC synthase (ACS) and ACC to ethylene by ACC oxidase (ACO). Additionally, *ERFs* are critical downstream components in ethylene signaling pathway (13). It was reported that the accumulation of endogenous ethylene can suppress *ACS* expression, and the transcript abundance of genes coding ethylene synthesis-related enzyme regulated their enzymic activities, influencing the production of ethylene. Thus, the downregulation of two *ACS* genes and upregulation of one *ACO* gene, was coincident with the performance of higher ethylene production in ultrasound treated strawberries within first 12 h of storage.

Transcription factors (TFs), are DNA-binding proteins that can specifically bind to the promoter region of eukaryotic genes or interact with certain auxiliary regulators, thereby regulating the specific expression of plant genes (62). According to the differences of DNA binding domains, TFs are primarily classified into several families, including MYB, AP2/ERF, WRKY, NAC, MADS-box, Homeobox, GRAS, bZIP, bHLH, etc. (20, 62). WRKY TFs compose one of the largest TF families in plants (63). Differential expression analysis showed that seven WRKY TFs displayed dramatic shifts in their mRNA levels (Figure 8). A *WRKY33* showed reduced transcription at 0 h. At 12 h after ultrasound treatment, six WRKY TFs including two *WRKY29* genes and four *WRKY33* genes were significantly down-regulated. Prior researches have revealed the vital regulatory function of WRKY TFs in response to biotic or abiotic stress (64). The differential expression of WRKY TFs indicated that *WRKY29* and *WRKY33* may play an important regulatory role in response to ultrasound treatment within 12 h.

### qRT-PCR analysis

**Figure 9:**
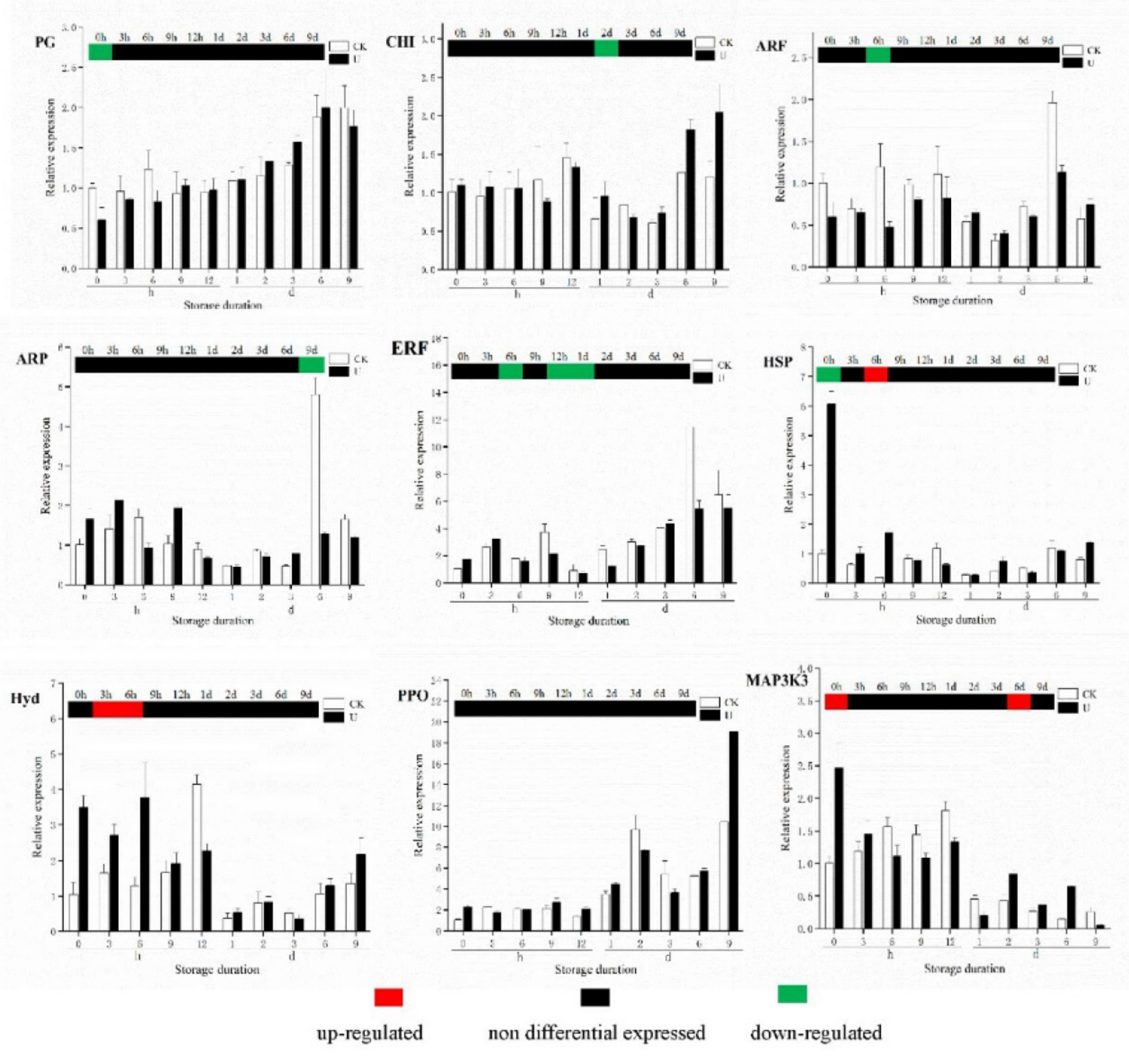
qRT-PCR validation of selected genes in strawberry after treatments. The relative expression of each gene was calculated based on its expression in the CK_0h sample; the columns was the qRT-PCR results of the selected gene, and the heat map above the columns was the RNA-seq results.

To validate the transcriptome sequencing data, nine candidate genes were randomly selected for qRT-PCR verification (Figure 9). The genes included one gene related to ROS metabolism pathway, three genes involved in auxin and ethylene signaling pathway, one gene related to MAPK signaling pathway, involved in fruit softening, and two genes involved in physiological metabolism of strawberry. For the nine genes, the results of change with storage time, and difference between two groups in gene expression obtained by qRT-PCR, were consistent with the results obtained from transcriptome sequencing analysis.

## Discussion

### Effect of ultrasound treatment on physiological characteristics and transcriptome process related to strawberry fruit postharvest softening

Softening is a vital factor that causes the deterioration of strawberry fruit quality. In this study, the firmness of postharvest strawberry fruit decreased gradually with storage time. However, ultrasound treatment significantly inhibited the firmness loss in the strawberry fruit during low temperature storage, accompanied with decreased SP content, PE and PG activity after 9 d of storage (Figure 2). Similar results were also observed in previous studies on ultrasound treated strawberry fruit (46, 59, 65, 66), and other fruit species, such as tomato (34, 35), cherry (67), and jujube (68). The inactivation effect of ultrasound on PE and PG has been studied to some extent, Raviyan et al. (69) found that the inactivation of PE in tomato increased with cavitation intensity, and similar study on PG indicated that high-intensity and prolonged ultrasound (22 kHz, >8.1 W·ml^−1^, >35 min) could induce the PG inactivation process (41). Explanations for PE, PG inactivation could be that free radicals and intense shear forces generated from extreme ultrasound conditions led to enzyme structural changes, and meanwhile changed the reactions with substrates and their surroundings (29, 41). Application of relatively low-intensity ultrasound (28 kHz, 0.15 W/cm^2^, 3 min) in this work, similarly, suppressed the activity levels of PG and PE. This result might be possible that inactivation effect on PE and PG of different ultrasound intensity varies from fruit species, and is influenced by regulation of gene expression and plant hormone in living plant.

ROS and ET, two kinds of important signal molecules, were investigated in this work. Our results indicated that ultrasound-treated strawberries showed lower H_2_O_2_ levels than the control within the initial 3 h and after day 18 of storage, although the H_2_O_2_ level was higher on days 2 and 6 (Figure 4A). As an important component of ROS signal, we speculated that ultrasound activated ROS metabolism to reduce H_2_O_2_, subsequently regulated the downstream response to ultrasound. Moreover, ultrasound significantly inhibited the production of ethylene, thereby delaying the softening and senescence of strawberry fruit (Figure 4B). Notably, ethylene production was higher in strawberry fruit treated with ultrasound during the first 6 d, this could be explained that ethylene production is one of the most common and marked reactions to abiotic stress abiotic stress in plant biology (70). The result of ethylene production was similar to previous report claiming that higher level of ethylene was observed in ultrasound-treated carrots (71).

According to transcriptomic results, changes in gene expression of several plant signaling pathway and TFs family were detected. In ROS metabolism and signaling pathway, gene expression related to ROS production was down-regulated, meanwhile gene expression linked to ROS scavenging was up-regulated at 0, 6, and 12 h, which indicated the inhibition of excessive ROS production and enhancement of ROS scavenging during the first 12 h (Figure 8). The results of ROS gene expression were aligned with the physiological response that H_2_O_2_ content of ultrasound-treated strawberries was lower within the initial 3 h of storage. The upregulation of ROS scavenging genes in ultrasound treated strawberry, was similar to the finding that DEGs encoding ROS scavenging enzyme GST were up-regulated in potato *in vitro* exposed to piezoelectric ultrasound (44). The differential expression of JA related genes suggests that there was an apparent response to ultrasound in JA metabolism and signaling pathway during the first 12 h, the release of JA was activated at 0 and 12 h while suppressed momently at 6 h. As shown in Figure 8, the general up-regulation of ethylene-related genes started at 12 h in the control strawberries, while started by 2 d in ultrasound-treated strawberries, which may cause to the relatively higher ethylene production of ultrasound-treated strawberries between days 2 and 6. These results suggest that the ultrasound treatment resulted in an earlier increase in ethylene production, which is associated closely with the regulation that ET metabolism and signaling pathway was activated until day 2. Interestingly, changes in expression profiles of auxin-related genes were also detected during the first 12 h, but the response was too complicated to explained now. In addition, we also found the differential expression of TFs genes including WRKY and ERF. *WRKY33* and *WRKY29* genes underwent strong downregulation in the initial 12 h, suggesting that *WRKY33* and *WRKY29* may play an important role in regulating downstream signaling and metabolic genes.

### Physiological mechanisms of ultrasound treatment inhibiting strawberry fruit postharvest softening

In fact, as reported in numerous studies, there are extremely complicated interactions in phytohormone, TFs and cell wall degrading enzymes.

The earlier change in ROS (H_2_O_2_) content can be considered as an immediate response took place right after ultrasound application (71). Acting as a signaling molecule, H_2_O_2_ may activate JA biosynthesis and signaling pathway (72). H_2_O_2_ and JA signals may work together for activating the subsequent responses, such as increased ethylene production as late response in ultrasound-treated carrot (71). There have been many studies demonstrated that ET can affect the gene expression of cell wall-degradation related enzymes in strawberry. With the application of ethylene, Villarreal et al. (27) found that *FaPG1, FaGal1*, and *FaGal2* were up-regulated, whereas *FaPME1, FaXyl1*, and *FaXTH1* were down-regulated, meanwhile *FaPLa* did not show any change at all in strawberry. Others reported the downregulation of *FaPG2* and *FaPLa*, the upregulation of *FaPE1*, and no changes in *FaPG1* in ethylene treated strawberry (73). In present study, the ethylene production of ultrasound-treated strawberries was higher than the control during 2–6 d, although the differential expression of *PE* and *PG*, the enzyme activity of PE and PG were markedly lower than the control after 9 d. This could be caused by the influence of ultrasound on the structure of the existing PE PG enzymes in strawberries.

On the other hand, TFs including WRKY, ERF, NAC, and MYB family genes can regulate phytohormone biosynthesis genes and signal transduction (38). ERFs, act as a key regulator, integrate the ET, ABA, JA, and redox signaling in plant, and conversely, JA and ABA have also been reported to participate in the regulation of ERFs under abiotic stresses (74, 75). Moreover, WRKY TFs are reported to be involved in JA mediated resistance (3). Eulgem and Somssich (76) reported that *WRKY33* can be activated by MAPK cascade pathway in Arabidopsis thaliana. However, the KEGG enrichment analysis revealed that MAPK signaling pathway was downregulated by ultrasound, which could be the reason why *WRKY33* genes were downregulated within the first 12 h. Additionally, extensive studies have shown that ERF genes are involved in the regulation of cell wall-modifying genes and fruit softening. In papaya, *CpERF9* acts as a transcriptional repressor of *CpPME1/2* and *CpPG5* (77). In peach, *PpeERF2* can suppress the expression of *PpePG1* by binding to its promotors (78).

**Figure 10:**
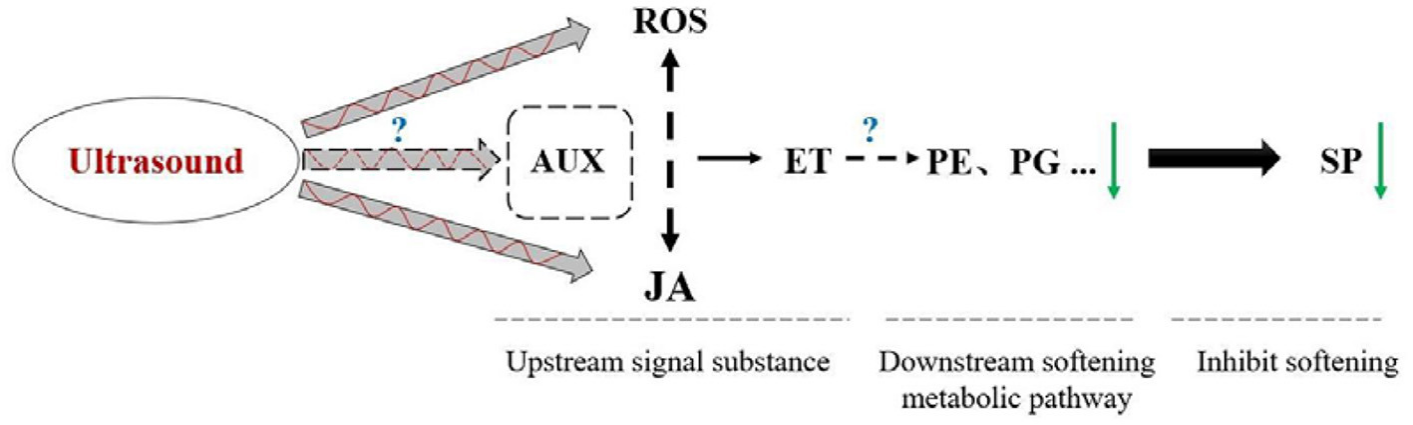
Hypothetical model explaining the physiological mechanisms inhibiting postharvest softening in strawberry treated with ultrasound. “+” presented promotion; “–” presented suppression; solid arrow presented direct regulation; and dotted arrow presented unclear regulation mechanism.

Based on the results presented in present work and prior researches, the mechanism ultrasound treatment inhibiting strawberry fruits postharvest softening was shown as Figure 10. After ultrasound treatment, the elimination of ROS (H_2_O_2_) and the release of JA signal was enhanced in the early stage (within 12 h). Under the effect of ROS and JA signal, ethylene signaling pathway was activated later (1.5 d later, at 2 d), resulting in longer release time of ET signal during the early middle stage (2–6 d), which is a likely important process for decreased PE and PG capacity in late storage time (after 9 d), inhibiting strawberry fruit postharvest softening ultimately (18–24 d). Herein, the transcription factors *WRKY29* and *WRKY33* may also play an important regulatory role. For further study, the hypothetical mechanism remains to be elucidated, through a chemical biology approach by using activators or inhibitors of the targeted signal molecules.

## Conclusion

Postharvest softening in strawberry fruits was effectively inhibited by ultrasound treatment. Ultrasound maintained the firmness and reduced the production of soluble pectin by retarding and suppressing the activities of pectin-degrading enzymes such as PE and PG, and therefore helping to keep textural quality of strawberry. Moreover, the content of signal substances hydrogen peroxide (H_2_O_2_) and ethylene had response to ultrasound treatment in early and late storage stage. Transcriptomic analysis revealed that ultrasound significantly changed the expression of genes related to ROS, ET, JA as well as other hormone signaling pathways and WRKY family including *RBOH, LOX, PPO, POD, ACS1_2_6, ACO, ERF, JAZ, IAA, SAUR, ARF*, together with *WRKY29* and *WRKY33* etc. A hypothetical mechanism model for the postharvest softening inhibition in strawberry by ultrasound treatment was proposed based on physiological and transcriptomic results of present study, as well as evidence from previous reports.

## Data availability statement

The data presented in the study are deposited in the NCBI repository, accession numbers BioProject ID PRJNA895742 and SRA ID SRP405228.

## Author contributions

All authors listed have made a substantial, direct, and intellectual contribution to the work and approved it for publication.

## Conflict of interest

The authors declare that the research was conducted in the absence of any commercial or financial relationships that could be construed as a potential conflict of interest.

## Publisher's note

All claims expressed in this article are solely those of the authors and do not necessarily represent those of their affiliated organizations, or those of the publisher, the editors and the reviewers. Any product that may be evaluated in this article, or claim that may be made by its manufacturer, is not guaranteed or endorsed by the publisher.

## Abbreviations

ACO: 1-aminocyclopropane-1-carboxylate acid oxidase
ACS: 1-aminocyclopropane-1-carboxylate acid synthase
ARF: auxin response factor
cDNA: Complementary DNA
DEGs: differentially expressed genes
FC: fold change
GO: Gene Ontology
JAZ: JASMONATE ZIM-domain protein
KEGG: Kyoto Encyclopedia of Genes and Genomes
LOX: Lipoxygenase
MAPK: Mitogen activated protein kinase
NR: Non-Redundant Protein Sequence Database
NCBI: National Center for Biotechnology Information
PE: pectin methylesterase
PG: polygalacturonase
qRT-PCR: real-time quantitative reverse transcription polymerase chain reaction
ROS: Reactive oxygen species
TF: Transcription factor
RNA-seq: RNA sequencing
NCED: 9-cis-epoxy carotenoid dioxygenase.

## Appendix A

**Table A1:** Primers used for qRT-PCR in strawberry fruits.

**Gene ID**	**Gene name**	**Sequences**	
*FxaC_27g39130*	*HSP*	F	TCTTCGACCCATTTTCCCTC
		R	CAGCTCAACCTTCACTTCTTCC
*FxaC_6g03070*	*Hyd*	F	AGTTTCCCTTTTGGTTGTGC
		R	CGTCCCTGGATACTCTCTGTG
*FxaC_4g12140*	*PG*	F	CCAGTAACTTCACGCTTTTTCTC
		R	TCCTAACCGCTCTCTCCCTC
*FxaC_11g01590*	*PPO*	F	CGAGGAGCAAGAATGAAAAGG
		R	GCGGCAACAACACCAAGC
*FxaC_1g11640*	*CHI*	F	TCGGCAGAACAATGCGGTAGA
		R	TTGGGGTTGGTGATGAGGGAG
*FxaC_1g17390*	*MAP3K3*	F	CCATCTACAACCTATGCCTCTA
		R	TGGGAATCATCACTGAACTATC
*FxaC_26g07610*	*ERF027*	F	GATACGGCCCTCAATTTCCCC
		R	TCCACATTTTCAACCCCACCC
*FxaC_8g31020*	*ARF*	F	TAACCAACAGGAACAGAGAATAATG
		R	CAAAAGAGGAGGATGTGAAATACCA
*FxaC_9g45470*	*ARP*	F	TGGAGAGACGAGGAGCAAGC
		R	ACGACGAACCCTATTCACAAAAC

The raw Illumina mRNA-seq data were submitted to NCBI and the processed data were deposited under GEO ID GSE123176, GSE135294, BioProject ID PRJNA507769, PRJNA558262, PRJNA895742 and SRA ID SRP171630, SRP217133.

## References

[1] Giampieri F Forbes-Hernandez TY Gasparrini M Alvarez-Suarez JM Afrin S Bompadre S. Strawberry as a health promoter: an evidence based review. Food Funct. (2015) 6:1386–98. 10.1039/C5FO00147A 25803191

[2] Wang F Xiao J Zhang Y Li R Liu L Deng J. Biocontrol ability and action mechanism of Bacillus halotolerans against Botrytis cinerea causing grey mould in postharvest strawberry fruit. Postharvest Biol Tec. (2021) 174:456. 10.1016/j.postharvbio.2020.111456

[3] Ji N Wang J Zuo X Li Y Li M Wang K. PpWRKY45 is involved in methyl jasmonate primed disease resistance by enhancing the expression of jasmonate acid biosynthetic and pathogenesis-related genes of peach fruit. Postharvest Biol Tec. (2021) 172:390. 10.1016/j.postharvbio.2020.111390

[4] Ji Y Hu W Liao J Xiu Z Jiang A Guan Y. Ethanol vapor delays softening of postharvest blueberry by retarding cell wall degradation during cold storage and shelf life. Postharvest Biol Tec. (2021) 177:538. 10.1016/j.postharvbio.2021.111538

[5] Moya-León MA Mattus-Araya E Herrera R. Molecular events occurring during softening of strawberry fruit. Front Plant Sci. (2019) 10:615. 10.3389/fpls.2019.00615 31156678 PMC6529986

[6] Rosli HG Civello PM Martínez GA. Changes in cell wall composition of three *Fragaria* × *ananassa* cultivars with different softening rate during ripening. PPB. (2004) 42:823–31. 10.1016/j.plaphy.2004.10.002 15596102

[7] Vicente AR Saladié M Rose JK Labavitch JM. The linkage between cell wall metabolism and fruit softening: looking to the future. J Sci Food Agr. (2007) 87:1435–48. 10.1002/jsfa.2837

[8] Xue C Guan SC Chen JQ Wen CJ Cai JF Chen X. Genome wide identification and functional characterization of strawberry pectin methylesterases related to fruit softening. BMC Plant Biol. (2020) 20:13. 10.1186/s12870-019-2225-9 31914938 PMC6950920

[9] Voragen AGJ Coenen G-J Verhoef RP Schols HA. Pectin, a versatile polysaccharide present in plant cell walls. Struct Chem. (2009) 20:263. 10.1007/s11224-009-9442-z26208585

[10] Dalagnol LMG Silveira VCC da Silva HB Manfroi V Rodrigues RC. Improvement of pectinase, xylanase and cellulase activities by ultrasound: Effects on enzymes and substrates, kinetics and thermodynamic parameters. Process Biochem. (2017) 61:80–7. 10.1016/j.procbio.2017.06.029

[11] Scheller HV Ulvskov P. Hemicelluloses. Annu Rev Plant Biol. (2010) 61:263–89. 10.1146/annurev-arplant-042809-112315 20192742

[12] Paniagua C Santiago-Doménech N Kirby AR Gunning AP Morris VJ Quesada MA. Structural changes in cell wall pectins during strawberry fruit development. PPB. (2017) 118:55–63. 10.1016/j.plaphy.2017.06.001 28618373

[13] He Y Xue J Li H Han S Jiao J Rao J. Ethylene response factors regulate ethylene biosynthesis and cell wall modification in persimmon (*Diospyros kaki* L.) fruit during ripening. Postharvest Biol Tec. (2020) 168:255. 10.1016/j.postharvbio.2020.111255

[14] Wang DD Yeats TH Uluisik S Rose JKC Seymour GB. Fruit softening: revisiting the role of pectin. Trends Plant Sci. (2018) 23:302–10. 10.1016/j.tplants.2018.01.006 29429585

[15] Marín-Rodríguez MC Orchard J Seymour GB. Pectate lyases, cell wall degradation and fruit softening. J Exp Bot. (2002) 53:2115–9. 10.1093/jxb/erf089 12324535

[16] Uluisik S Seymour GB. Pectate lyases: their role in plants and importance in fruit ripening. Food Chem. (2020) 309:125559. 10.1016/j.foodchem.2019.125559 31679850

[17] Wang K Li T Chen S Li Y Rashid A. The biochemical and molecular mechanisms of softening inhibition by chitosan coating in strawberry fruit (*Fragaria* × *ananassa*) during cold storage. Sci Hortic-Amsterdam. (2020) 271:483. 10.1016/j.scienta.2020.109483

[18] Apel K Hirt H. Reactive oxygen species: metabolism, oxidative stress, and signal transduction. Annu Rev Plant Biol. (2004) 55:373–99. 10.1146/annurev.arplant.55.031903.141701 15377225

[19] McAtee P Karim S Schaffer R David K. A dynamic interplay between phytohormones is required for fruit development, maturation, and ripening. F PLS. (2013) 4:79. 10.3389/fpls.2013.00079 23616786 PMC3628358

[20] Tang N An J Deng W Gao Y Chen Z Li Z. Metabolic and transcriptional regulatory mechanism associated with postharvest fruit ripening and senescence in cherry tomatoes. Postharvest Biol Tec. (2020) 168:274. 10.1016/j.postharvbio.2020.111274

[21] Huaiyu Z Fangrui L Junjie W Qingrong Y Peng W Huijun Z. Salicylic acid inhibits the postharvest decay of goji berry (*Lycium barbarum* L.) by modulating the antioxidant system and phenylpropanoid metabolites. Postharvest Biol Tec. (2021) 178:111558. 10.1016/j.postharvbio.2021.111558

[22] Jia H-F Chai Y-M Li C-L Lu D Luo J-J Qin L. Abscisic acid plays an important role in the regulation of strawberry fruit ripening. Plant Physiol. (2011) 157:188–99. 10.1104/pp.111.177311 21734113 PMC3165869

[23] Kou X Yang S Chai L Wu C Zhou J Liu Y. Abscisic acid and fruit ripening: Multifaceted analysis of the effect of abscisic acid on fleshy fruit ripening. Sci Hortic-Amsterdam. (2021) 281:109999. 10.1016/j.scienta.2021.109999

[24] Symons GM Chua YJ Ross JJ Quittenden LJ Davies NW Reid JB. Hormonal changes during non-climacteric ripening in strawberry. J Exp Bot. (2012) 63:4741–50. 10.1093/jxb/ers147 22791823 PMC3428006

[25] Zhou Q Zhang F Ji S Dai H Zhou X Wei B. Abscisic acid accelerates postharvest blueberry fruit softening by promoting cell wall metabolism. Sci Hortic-Amsterdam. (2021) 288:110325. 10.1016/j.scienta.2021.110325

[26] Kou J Wei C Zhao Z Guan J Wang W. Effects of ethylene and 1-methylcyclopropene treatments on physiological changes and ripening-related gene expression of ‘Mopan' persimmon fruit during storage. Postharvest Biol Tec. (2020) 166:185. 10.1016/j.postharvbio.2020.111185

[27] Villarreal NM Marina M Nardi CF Civello PM Martínez GA. Novel insights of ethylene role in strawberry cell wall metabolism. Plant Sci. (2016) 252:1–11. 10.1016/j.plantsci.2016.06.018 27717444

[28] Han Y Chen C Yan Z Li J Wang Y. The methyl jasmonate accelerates the strawberry fruits ripening process. Sci Hortic-Amsterdam. (2019) 249:250–6. 10.1016/j.scienta.2019.01.061

[29] Islam MN Zhang M Adhikari B. The inactivation of enzymes by ultrasound—a review of potential mechanisms. Food Rev Int. (2014) 30:1–21. 10.1080/87559129.2013.85377217045799

[30] Pinheiro J Alegria C Abreu M Goncalves EM Silva CLM. Influence of postharvest ultrasounds treatments on tomato (*Solanum lycopersicum*, cv. Zinac) quality and microbial load during storage. Ultrason Sonochem. (2015) 27:552–9. 10.1016/j.ultsonch.2015.04.009 25922160

[31] Jiang Q Zhang M Xu B. Application of ultrasonic technology in postharvested fruits and vegetables storage: a review. Ultrason Sonochem. (2020) 69:105261. 10.1016/j.ultsonch.2020.105261 32702635

[32] Chemat F Zill EH Khan MK. Applications of ultrasound in food technology: Processing, preservation and extraction. Ultrason Sonochem. (2011) 18:813–35. 10.1016/j.ultsonch.2010.11.023 21216174

[33] Temizkan R Atan M Büyükcan MB Caner C. Efficacy evaluation of ultrasound treatment on the postharvest storability of white nectarine by both physicochemical and image processing analyses. Postharvest Biol Tec. (2019) 154:41–51. 10.1016/j.postharvbio.2019.04.014

[34] Alenyorege EA Ma H Ayim I Aheto JH Hong C Zhou C. Effect of multi-frequency multi-mode ultrasound washing treatments on physicochemical, antioxidant potential and microbial quality of tomato. J Food Meas Charact. (2018) 13:677–86. 10.1007/s11694-018-9980-4

[35] Lu C Ding J Park HK Feng H. High intensity ultrasound as a physical elicitor affects secondary metabolites and antioxidant capacity of tomato fruits. Food Control. (2020) 113:176. 10.1016/j.foodcont.2020.107176

[36] Ni Z Xu S Ying T. The effect and mechanism of ultrasonic treatment on the postharvest texture of shiitake mushrooms (*Lentinula edodes*). JFST. (2018) 53:1847–54. 10.1111/ijfs.13768

[37] Fan K Wu J Chen L. Ultrasound and its combined application in the improvement of microbial and physicochemical quality of fruits and vegetables: a review. Ultrason Sonochem. (2021) 80:105838. 10.1016/j.ultsonch.2021.105838 34801817 PMC8605411

[38] Xu F Liu S Xiao Z Fu L. Effect of ultrasonic treatment combined with 1-methylcyclopropene (1-MCP) on storage quality and ethylene receptors gene expression in harvested apple fruit. J Food Biochem. (2019) 43:e12967. 10.1111/jfbc.12967 31368577

[39] Huang G Chen S Dai C Sun L Sun W Tang Y. Effects of ultrasound on microbial growth and enzyme activity. Ultrason Sonochem. (2017) 37:144–9. 10.1016/j.ultsonch.2016.12.018 28427617

[40] Larsen LR van der Weem J Caspers-Weiffenbach R Schieber A Weber F. Effects of ultrasound on the enzymatic degradation of pectin. Ultrason Sonochem. (2021) 72:105465. 10.1016/j.ultsonch.2021.105465 33497958 PMC7838710

[41] Ma X Wang W Zou M Ding T Ye X Liu D. Properties and structures of commercial polygalacturonase with ultrasound treatment: role of ultrasound in enzyme activation. RSC Adv. (2015) 5:107591–600. 10.1039/C5RA19425C

[42] Dobránszki J Hidvégi N Gulyás A Teixeira da Silva JA. mRNA transcription profile of potato (*Solanum tuberosum* L.) exposed to ultrasound during different stages of in vitro plantlet development. Plant Mol Biol. (2019) 100:511–25. 10.1007/s11103-019-00876-0 31037600 PMC6586710

[43] Dobránszki J Hidvégi N Gulyás A Tóth B Teixeira da Silva JA. Abiotic stress elements in *in vitro* potato (*Solanum tuberosum* L.) exposed to air-based and liquid-based ultrasound: a comparative transcriptomic assessment. Prog Biophys Mol Bio. (2020) 158:47–56. 10.1016/j.pbiomolbio.2020.09.001 32916176

[44] Teixeira da Silva JA Hidvégi N Gulyás A Tóth B Dobránszki J. Transcriptomic response of *in vitro* potato (*Solanum tuberosum* L.) to piezoelectric ultrasound. Plant Mol Biol Rep. 38:404–18. 10.1007/s11105-020-01204-3

[45] Ghosh R Mishra RC Choi B Kwon YS Bae DW Park S-C. Exposure to sound vibrations lead to transcriptomic, proteomic and hormonal changes in arabidopsis. Sci Rep. (2016) 6:37484. 10.1038/srep37484 27883000 PMC5122249

[46] Cao S Hu Z Pang B Wang H Xie H Wu F. Effect of ultrasound treatment on fruit decay and quality maintenance in strawberry after harvest. Food Control. (2010) 21:529–32. 10.1016/j.foodcont.2009.08.002

[47] Vicente AR Costa ML Martínez GA Chaves AR Civello PM. Effect of heat treatments on cell wall degradation and softening in strawberry fruit. Postharvest Biol Tec. (2005) 38:213–22. 10.1016/j.postharvbio.2005.06.005

[48] Karakurt Y Huber DJ. Activities of several membrane and cell-wall hydrolases, ethylene biosynthetic enzymes, and cell wall polyuronide degradation during low-temperature storage of intact and fresh-cut papaya (Carica papaya) fruit. Postharvest Biol Tec. (2003) 28:219–29. 10.1016/S0925-5214(02)00177-1

[49] Santos JG Fernandes FAN de Siqueira Oliveira L de Miranda MRA. Influence of ultrasound on fresh-cut mango quality through evaluation of enzymatic and oxidative metabolism. FABT. (2015) 8:1532–42. 10.1007/s11947-015-1518-8

[50] Brennan T Frenkel C. Involvement of hydrogen peroxide in the regulation of senescence in pear. Plant Physiol. (1977) 59:411–6. 10.1104/pp.59.3.411 16659863 PMC542414

[51] Mditshwa A Fawole OA Vries F van der Merwe K Crouch E Opara UL. Repeated application of dynamic controlled atmospheres reduced superficial scald incidence in ‘Granny Smith' apples. Sci Hortic-Amsterdam. (2017) 220:168–75. 10.1016/j.scienta.2017.04.003

[52] Edger PP Poorten TJ VanBuren R Hardigan MA Colle M McKain MR. Origin and evolution of the octoploid strawberry genome. Nat Genet. (2019) 51:541–7. 10.1038/s41588-019-0356-4 30804557 PMC6882729

[53] Kim D Langmead B Salzberg SL. HISAT: a fast spliced aligner with low memory requirements. Nat Med. (2015) 12:357–60. 10.1038/nmeth.3317 25751142 PMC4655817

[54] Li B Dewey CN. RSEM: accurate transcript quantification from RNA-Seq data with or without a reference genome. BMC Bioinformatics. (2011) 12:323. 10.1186/1471-2105-12-323 21816040 PMC3163565

[55] Love MI Huber W Anders S. Moderated estimation of fold change and dispersion for RNA-seq data with DESeq2. Genome Biol. (2014) 15:550. 10.1186/s13059-014-0550-8 25516281 PMC4302049

[56] Benjamini Y Hochberg Y. Controlling the false discovery rate: a practical and powerful approach to multiple testing. J R Stat Soc B Stat. (1995) 57:289–300. 10.1111/j.2517-6161.1995.tb02031.x

[57] Pfaffl MW. A new mathematical model for relative quantification in real-time RT-PCR. NAR. (2001) 29:e45. 10.1093/nar/29.9.e45 11328886 PMC55695

[58] Alexandre EMC Brandao TRS Silva CLM. Efficacy of non-thermal technologies and sanitizer solutions on microbial load reduction and quality retention of strawberries. J Food Eng. (2012) 108:417–26. 10.1016/j.jfoodeng.2011.09.002

[59] Gani A Baba WN Ahmad M Shah U Khan AA Wani IA. Effect of ultrasound treatment on physico-chemical, nutraceutical and microbial quality of strawberry. LWT-Food Sci Technol. (2016) 66:496–502. 10.1016/j.lwt.2015.10.067

[60] Langer SE Oviedo NC Marina M Burgos JL Martínez GA Civello PM. Effects of heat treatment on enzyme activity and expression of key genes controlling cell wall remodeling in strawberry fruit. Plant Physiol Bioch. (2018) 130:334–44. 10.1016/j.plaphy.2018.07.015 30053739

[61] Kwak JM Mori IC Pei ZM Leonhardt N Torres MA Dangl JL. NADPH oxidase AtrbohD and AtrbohF genes function in ROS-dependent ABA signaling in Arabidopsis. EMBO J. (2003) 22:2623–33. 10.1093/emboj/cdg277 12773379 PMC156772

[62] Yang S Zhou J Watkins CB Wu C Feng Y Zhao X. NAC transcription factors SNAC4 and SNAC9 synergistically regulate tomato fruit ripening by affecting expression of genes involved in ethylene and abscisic acid metabolism and signal transduction. Postharvest Biol Tec. (2021) 178:555. 10.1016/j.postharvbio.2021.111555

[63] Rushton PJ Somssich IE Ringler P Shen QJ. WRKY transcription factors. Trends Plant Sci. (2010) 15:247–58. 10.1016/j.tplants.2010.02.006 20304701

[64] Lai Z Vinod KM Zheng Z Fan B Chen Z. Roles of arabidopsisWRKY3 and WRKY4 transcription factors in plant responses to pathogens. BMC Plant Biol. (2008) 8:68. 10.1186/1471-2229-8-68 18570649 PMC2464603

[65] Aday MS Temizkan R Büyükcan MB Caner C. An innovative technique for extending shelf life of strawberry: ultrasound. LWT-Food Sci Technol. (2013) 52:93–101. 10.1016/j.lwt.2012.09.01332567052

[66] Zhao S Lai S Chen F Yang H. Combined effects of ultrasound and calcium on the chelate-soluble pectin and quality of strawberries during storage. Carbohyd Polym. (2018) 200:427–35. 10.1016/j.carbpol.2018.08.013 30177183

[67] Muzaffar S Ahmad M Wani SM Gani A Baba WN Shah U. Ultrasound treatment: effect on physicochemical, microbial and antioxidant properties of cherry (Prunus avium). J Food Sci Tech Mys. (2016) 53:2752–9. 10.1007/s13197-016-2247-3 27478231 PMC4951428

[68] Zhi H Liu Q Xu J Dong Y Liu M Zong W. Ultrasound enhances calcium absorption of jujube fruit by regulating the cellular calcium distribution and metabolism of cell wall polysaccharides. J Sci Food Agr. (2017) 97:5202–10. 10.1002/jsfa.8402 28447385

[69] Raviyan P Zhang Z Feng H. Ultrasonication for tomato pectinmethylesterase inactivation: effect of cavitation intensity and temperature on inactivation. J Food Eng. (2005) 70:189–96. 10.1016/j.jfoodeng.2004.09.028

[70] Surjadinata BB Jacobo-Velazquez DA Cisneros-Zevallos L. Physiological role of reactive oxygen species, ethylene, and jasmonic acid on UV light induced phenolic biosynthesis in wounded carrot tissue. Postharvest Biol Tec. (2021) 172:388. 10.1016/j.postharvbio.2020.111388

[71] Cuéllar-Villarreal MdR Ortega-Hernández E Becerra-Moreno A Welti-Chanes J Cisneros-Zevallos L Jacobo-Velázquez DA. Effects of ultrasound treatment and storage time on the extractability and biosynthesis of nutraceuticals in carrot (*Daucus carota*). Postharvest Bio Tec. (2016) 119:18–26. 10.1016/j.postharvbio.2016.04.013

[72] Wu J Ge X. Oxidative burst, jasmonic acid biosynthesis, and taxol production induced by low-energy ultrasound in Taxus chinensis cell suspension cultures. Biotechnol Bioeng. (2004) 85:714–21. 10.1002/bit.10911 14991649

[73] Merchante C Vallarino JG Osorio S Aragüez I Villarreal N Ariza MT. Ethylene is involved in strawberry fruit ripening in an organ-specific manner. J Exp Bot. (2013) 64:4421–39. 10.1093/jxb/ert257 24098047 PMC3808323

[74] Müller M Munné-Bosch S. Ethylene response factors: a key regulatory hub in hormone and stress signaling. Plant Physiol. (2015) 169:32–41. 10.1104/pp.15.00677 26103991 PMC4577411

[75] Oda S Sakaguchi M Yang X Liu Q Iwasaki K Nishibayashi K. Ultrasonic treatment suppresses ethylene signaling and prolongs the freshness of spinach. Food Chem. (2021) 2:100026. 10.1016/j.fochms.2021.100026 35415625 PMC8991814

[76] Eulgem T Somssich IE. Networks of WRKY transcription factors in defense signaling. Curr Opin Plant Biol. (2007) 10:366–71. 10.1016/j.pbi.2007.04.020 17644023

[77] Fu C-C Han Y-C Qi X-Y Shan W Chen J-Y Lu W-J. Papaya CpERF9 acts as a transcriptional repressor of cell-wall-modifying genes CpPME1/2 and CpPG5 involved in fruit ripening. Plant Cell Rep. (2016) 35:2341–52. 10.1007/s00299-016-2038-3 27502602

[78] Wang X Zeng W Ding Y Wang Y Niu L Yao J-L. Peach ethylene response factor PpeERF2 represses the expression of ABA biosynthesis and cell wall degradation genes during fruit ripening. Plant Sci. (2019) 283:116–26. 10.1016/j.plantsci.2019.02.009 31128681

